# Use of Bacteria- and Fungus-Binding Mesh in Negative Pressure Wound Therapy Provides Significant Granulation Tissue Without Tissue Ingrowth

**Published:** 2014-01-17

**Authors:** Malin Malmsjö, Sandra Lindstedt, Richard Ingemansson, Lotta Gustafsson

**Affiliations:** ^a^Departments of Ophthalmology, Lund University and Skåne University Hospital, Lund, Sweden; ^b^Cardiothoracic Surgery, Lund University and Skåne University Hospital, Lund, Sweden

**Keywords:** blood flow, experimental surgery, negative pressure wound therapy, wound contraction, wound dressing, wound healing

## Abstract

**Objective:** Bacteria- and fungus-binding mesh traps and inactivates bacteria and fungus, which makes it interesting, alternative, and wound filler for negative pressure wound therapy (NPWT). The aim of this study was to compare pathogen-binding mesh, black foam, and gauze in NPWT with regard to granulation tissue formation and ingrowth of wound bed tissue in the wound filler. **Methods:** Wounds on the backs of 8 pigs underwent 72 hours of NPWT using pathogen-binding mesh, foam, or gauze. Microdeformation of the wound bed and granulation tissue formation and the force required to remove the wound fillers was studied. **Results:** Pathogen-binding mesh produced more granulation tissue, leukocyte infiltration, and tissue disorganization in the wound bed than gauze, but less than foam. All 3 wound fillers caused microdeformation of the wound bed surface. Little force was required to remove pathogen-binding mesh and gauze, while considerable force was needed to remove foam. This is the result of tissue growth into the foam, but not into pathogen-binding mesh or gauze, as shown by examination of biopsy sections from the wound bed. **Conclusions:** This study shows that using pathogen-binding mesh as a wound filler for NPWT leads to a significant amount of granulation tissue in the wound bed, more than that with gauze, but eliminates the problems of ingrowth of the wound bed into the wound filler. Pathogen-binding mesh is thus an interesting wound filler in NPWT.

Negative pressure wound therapy (NPWT) employs a closed drainage system to apply controlled suction to a wound bed. NPWT has revolutionized the treatment of patients with both chronic and acute wounds,^1^^,^^2^ including orthopedic trauma,^3^ soft tissue trauma,^4^ skin grafts,^5^ pressure ulcers,^6^ venous leg ulcers,^7^ vascular surgery wounds, diabetic foot ulcers,^8^ burns,^9^ surgical infections,^10^ and the management of open wounds following abdominal surgery^11^ and thoracic surgery.^12^^,^^13^ We are beginning to understand the mechanisms by which negative pressure promotes wound healing. NPWT creates a moist environment,^14^ drains exudate,^15^^-^17 reduces tissue edema,^18^ contracts the wound edges,^15^^-^17 mechanically stimulates the wound bed,^19^^-^21 alters blood flow in the wound edges,^16^^,^^22^^-^24 and stimulates angiogenesis^25^^,^^26^ and the formation of granulation tissue.^16^ The biological effects of NPWT on the wound bed depend on the type of wound filler used and the negative pressure setting.

There is a common misconception that NPWT controls or reduces the bacterial burden in the wound. In an initial study on pig wounds inoculated with human *Staphylococcus aureus* and *Staphylococcus epidermidis* a reduction in bacterial counts during the course of NPWT was reported.^16^ However, no clinical studies since then have been able to confirm the early in vivo findings of Morykwas et al,^27^^-^30 and some have even reported an increase in bacterial numbers during NPWT.^27^^,^^31^^,^^32^ NPWT has been shown to cause a shift in the bacterial species toward biofilm-producing organisms such as *S. aureus* and *S. epidermidis*.^27^^,^^28^^,^^30^ It has been hypothesized that occlusion and negative pressure create relative hypoxia, thus promoting anaerobes and a shift in microorganism populations.^30^ Furthermore, the gauze used in NPWT has been a particular type of cotton gauze (Kerlix AMD), which may provide pathogen-binding control because it is impregnated with polyhexamethylene biguanide.^33^ However, the current recommendation is that NPWT should not be used in isolation to control wound infections (www.npwtexperts.com).

Studies are now emerging showing that the amount and character of granulation tissue formed differ depending on the type of wound filler used for NPWT. Foam and gauze are the most common wound filler materials used in NPWT. The use of foam produces thick granulation tissue,^19^^,^^34^^,^^35^ while gauze produces thinner but denser granulation tissue.^19^^,^^34^ The choice of wound filler for NPWT may therefore be tailored to the individual wound.^36^ Pathogen-binding mesh may provide an interesting alternative wound filler for NPWT. Mesh of this kind makes use of the hydrophobic interaction to remove pathogenic wound bacteria. The hydrophobic interaction is a basic physical phenomenon causing hydrophobic (water-repellent) particles to accumulate in an aqueous environment, held together by the forces of the surrounding water molecules. Bacteria have hydrophobic cell surface structures that allow them to adhere to wound tissue in the initial phase of infection. Pathogen-binding mesh is coated with a fatty acid derivative, which gives the dressing strongly hydrophobic properties. Wound bacteria are thus irreversibly bound to the dressing when they come into contact with the hydrophobic dressing fibers in the moist wound environment.^37^ Pathogen-binding mesh can thus adsorb and inactivate a wide range of bacteria, for example, *Staphylococcus aureus* and *Pseudomonas aeruginosa*, and fungi. It has been shown to reduce the microbial load in wounds^38^^,^^39^ and offers a nonallergic, nontoxic alternative for reducing the microbial load in open wounds. The mesh binds and inactivates bacteria and fungus without the development of resistance among microorganisms.

The aim of this study was to compare the effects of hydrophobic pathogen-binding mesh with foam and gauze during NPWT with regard to wound bed appearance and granulation tissue formation using an *in vivo* porcine wound model.

## MATERIAL AND METHODS

### Animals

Eight healthy domestic pigs of both sexes, with a mean body weight of 70 kg were used. The experimental protocol for this study was approved by the Ethics Committee for Animal Research, Lund University, Sweden. All animals received humane care in compliance with the European Convention on Animal Care.

### Anesthesia

The pigs were fasted overnight with free access to water. Premedication was performed with an intramuscular injection of xylazine (Rompun vet 20 mg/mL; Bayer AG, Leverkusen, Germany; 2 mg/kg) mixed with ketamine (Ketaminol vet 100 mg/mL; Farmaceutici Gellini S.p.A., Aprilia, Italy; 20 mg/kg). The animals were orally intubated with cuffed endotracheal tubes. Mechanical ventilation was established with a Siemens-Elema ventilator (Siemens-Elema AB, Solna, Sweden) in the volume-controlled mode (65% nitrous oxide, 35% oxygen). Ventilatory settings were identical for all animals (respiratory rate, 15 breaths per minutes; minute ventilation, 12 L/min). A positive end-expiratory pressure of 5-cm H_2_O was applied. Two peripheral veins in the pig's ear were cannulated for the induction and maintenance of anesthesia and for fluid administration. Anesthesia was maintained with a continuous infusion of ketamine (Ketaminol vet 50 mg/mL; Farmaceutici Gellini S.p.A., Aprilia, Italy; 0.4–0.6 mg/kg/h). Complete neuromuscular blockade was achieved with a continuous infusion of pancuronium bromide (Pavulon; N.V. Organon, OSS, the Netherlands; 0.3–0.5 mg/kg/h). Fluid loss was compensated by continuous infusion of Ringer's acetate at a rate of 200 mL/kg/h for the first 24 hours, followed by 110 mL/h for the remainder of the experiment. The animals received total parenteral nutrition (Kabiven; Fresenius Kabi AB, Uppsala, Sweden). Antibiotics were given once daily as intravenous bolus injections (Streptocillin vet 250 mg/mL + 200 mg/mL; Boehringer Ingelheim Vetmedica, Malmö, Sweden; 10 mL). A Foley catheter was inserted into the urinary bladder through a suprapubic cystostomy. After the experiments were completed, the animals were euthanized with a lethal dose (60 mmol) of intravenous potassium chloride.

### Negative pressure wound therapy

Circular wounds, 6 cm in diameter, extending into the subcutaneous tissue, were created on each pig's back. The wounds were filled with saline-moistened AMD gauze (Kendall Kerlix AMD, Tyco Healthcare Group, Mansfield, Massachusetts), hydrophobic pathogen binding mesh (Sorbact, Abigo Medical AB, Gothenburg, Sweden), or black polyurethane foam with an open cell structure (VAC Granufoam, KCI, San Antonio, Texas). The drainage tube was placed on the top of the wound filler, imbedded in adhesive drape and connected to a vacuum source.

### Quantity of granulation tissue formed

The quantity of granulation tissue formed during NPWT was graded by 2 different surgeons, as described in a previous study.^40^ The scale ranged from 0 (a pale wound bed without granulation) to 5 (fully granulated tissue and vascularized wound bed). The surgeons who performed the grading observed the wound bed after all the NPWT dressings had been removed, and thus had no knowledge of which dressing had been used. Grading was performed separately by each surgeon.

### Force measurements

After 72 hours of NPWT (as described earlier), the adhesive film dressing covering the wound was cut along the borderline between the tissue and the wound filler, and the drain was cut off. The wound filler was attached to a custom-made force measurement device and withdrawn at a constant speed of 4 mm/s.^40^ The force required to remove the wound filler was plotted as a function of time using a computer.

### Histological examination

A strip of the wound filler material (1 × 1 × 2 cm^3^) was sutured onto the bottom of each wound. After NPWT, the strip and the underlying wound bed tissue were excised with a scalpel. The tissue was then treated in 4% paraformaldehyde, dehydrated and finally embedded in paraffin, and left overnight. Biopsies were sectioned (4-μm thick), using a rotary microtome (HM 355, ThermoFisher Scientific, Massachusetts), mounted on glass slides, and stained using hematoxylin-eosin staining.

### Characteristics of the granulation tissue formed

Biopsy sections were evaluated with regard to microdeformation (ie, wound bed surface undulations), ingrowth into the wound filler, and morphology of the underlying tissue, including disorganization of the cells in the wound bed (ie, disruption of the contacts between the cells and differences in cell size), and leukocyte count (number per μm^2^).

### Limitations

Reduction in bacterial load by pathogen binding mesh has been shown in previous studies^39^^,^^41^ and was not the scope of this study. The aim of the present study was to determine the suitability for pathogen-binding mesh to be used for NPWT with regard to its physiological properties, compared to presently used wound fillers for NPWT. It has clearly been shown that the acute wound model in the pig is well suited for studying the physiological properties of wound fillers.^42^ When these pig wounds have been inoculated with bacteria to study the effects of NPWT on bacterial burden, the results have not been reliable.^43^

### Calculations and statistics

Calculations were performed using GraphPad 5.0 software (San Diego, California). Statistical analysis was performed using the Mann-Whitney test when comparing 2 groups, and the Kruskal-Wallis test with Dunn's posttest for multiple comparisons when comparing 3 groups or more. Significance was defined as *P* < .05. All differences referred to in the text were statistically significant. Results are presented as the means of 8 measurements ± the standard error of the mean.

## RESULTS

### Quantity and characteristics of granulation tissue

**Figure 1:**
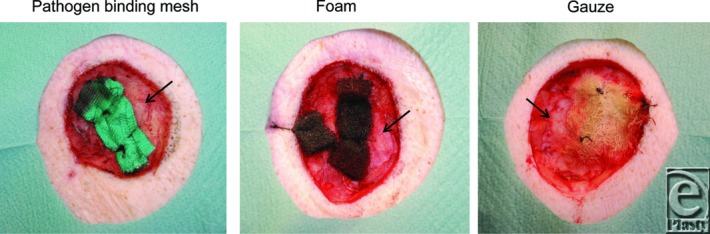
Representative photos of the wound bed after treatment with a negative pressure of −120 mm Hg using pathogen binding mesh, foam, or gauze for 72 hours. Note that the granulation tissue formed under the pathogen-binding mesh has a structure between that of those formed under foam and gauze.

**Figure 2:**
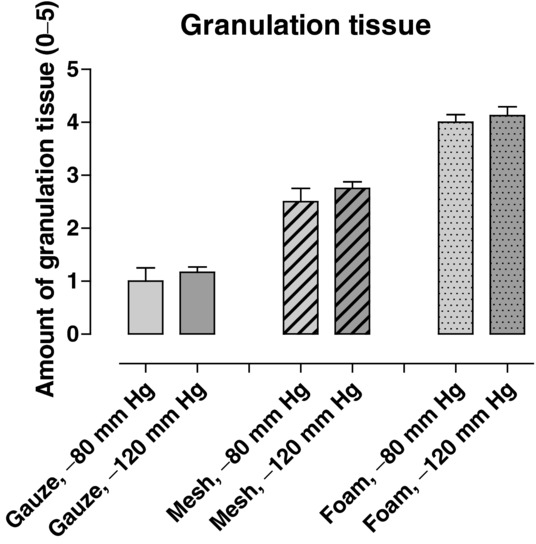
The amount of granulation tissue formed after 72 hours of NPWT at −80 and −120 mm Hg, using pathogen-binding mesh, foam, or gauze. The amount of granulation tissue was graded on a scale from 0 to 5 by 2 different surgeons. Grading was performed blinded, and separately by each surgeon. Results are shown as means ± standard error of the mean of 8 experiments. NPWT indicates negative pressure wound therapy.

**Figure 3:**
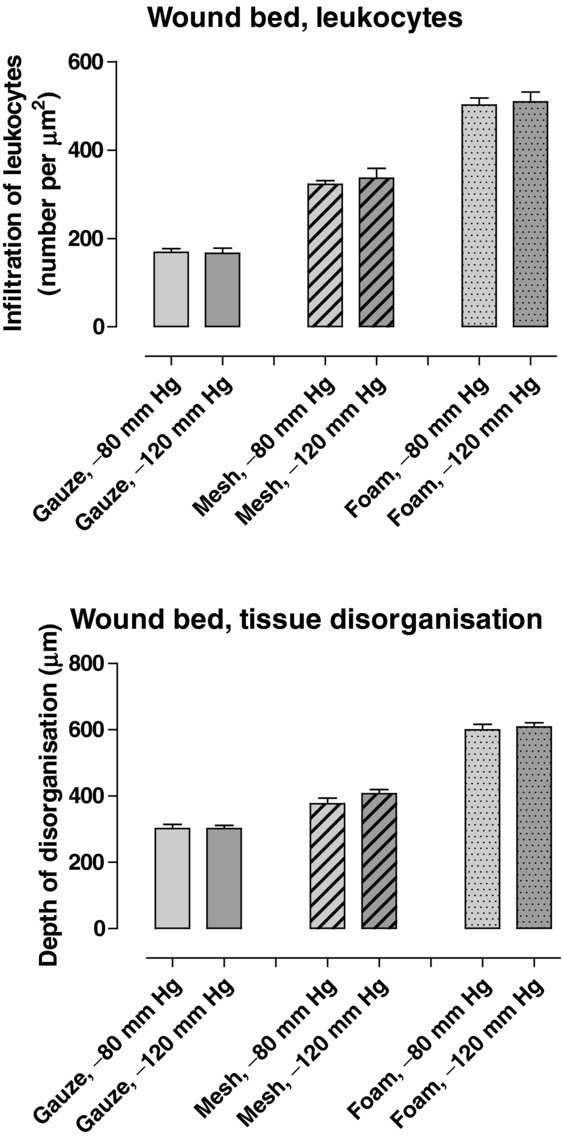
The number of leukocytes per μm^2^ and the depth of tissue disorganization in tissue samples from wound beds treated for 72 hours with NPWT at −120 mm Hg using pathogen-binding mesh, foam, or gauze (means ± standard error of the mean). It can be seen that the degree of leukocyte infiltration and tissue disorganization under pathogen-binding mesh are between those resulting from the use of gauze and foam. NPWT indicates negative pressure wound therapy.

Pathogen-binding mesh led to the formation of more granulation tissue than gauze, but less than foam (Figs 1 and 2). The wound bed characteristics, that is, the tissue morphology and the cellular infiltrate in the wound bed underlying the wound filler, were examined histologically. Pathogen-binding mesh was found to lead to more leukocyte infiltration and tissue disorganization, that is, the disruption of contacts between the cells and differences in cell size, in the wound bed than gauze, but less than foam (Fig 3).

### Microdeformation

**Figure 4:**
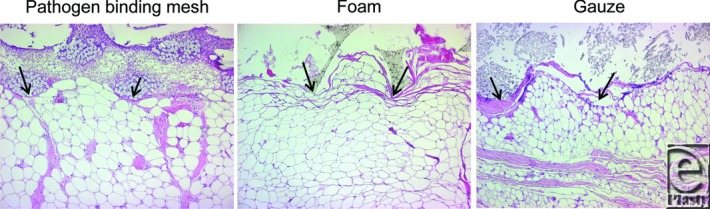
Hematoxylin-eosin–stained sections of biopsies from wound beds treated for 72 hours with NPWT at −120 mm Hg using pathogen-binding mesh, foam, or gauze. The wound filler is seen at the top of the images and the tissue (mainly composed of adipocytes) at the bottom. All wound fillers cause a repeating pattern of wound surface undulations. Small tissue blebs can be seen in the pores of the foam and the spaces between the threads in the gauze. The intrusion of tissue into the filler is indicated by the arrows. NPWT indicates negative pressure wound therapy.

Pathogen-binding mesh, foam, and gauze compressed the wound bed so that small tissue blebs were drawn into the empty spaces of the gauze and the pores of the foam, as indicated by the arrows in Figure 4.

### Ingrowth and the force required to remove the wound filler

**Figure 5:**
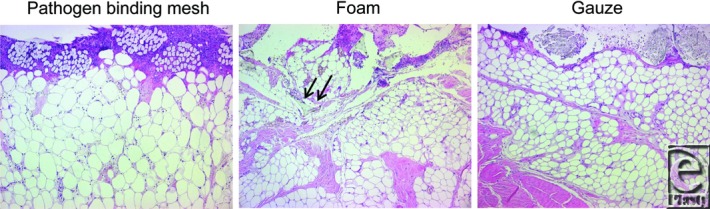
Hematoxylin-eosin–stained sections of biopsies from wound beds after 72 hours of NPWT at −120 mm Hg using pathogen-binding mesh, foam, or gauze. The ingrowth of tissue into the foam is indicated by arrows. No such ingrowth can be seen in the pathogen-binding mesh, or gauze. NPWT indicates negative pressure wound therapy.

**Figure 6:**
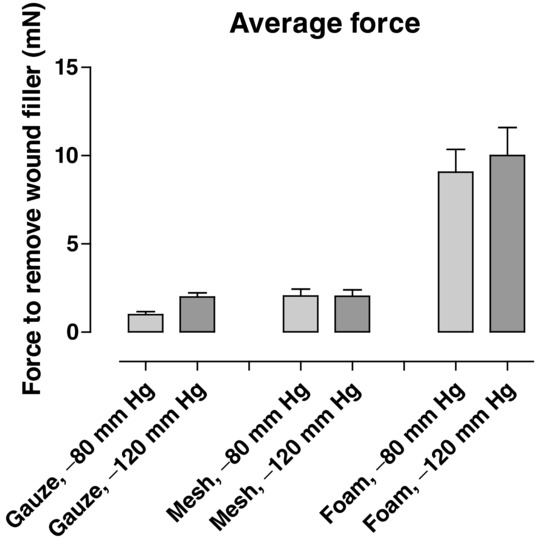
Force required to remove pathogen-binding mesh, foam, and gauze after 3 days of NPWT at −80 and −120 mm Hg. The average force was calculated over time. The results are shown as means ± standard error of the mean. It can be seen that more force is needed to remove foam than the pathogen-binding mesh or gauze. NPWT indicates negative pressure wound therapy.

The wound bed tissue grew into foam (643.7 ± 22.0 μm), but not into pathogen-binding mesh or gauze (Fig 5). Little force was required to remove the pathogen-binding mesh (2.1 ± 0.4 mN) and gauze (1.0 ± 0.2 mN), while considerable force was needed to remove the foam (9.1 ± 1.3 mN, Fig 6).

## DISCUSSION

### Granulation tissue formation

Pathogen-binding mesh led to the production of more granulation tissue than gauze, but less than foam. Likewise, the use of pathogen-binding mesh induced more leukocyte infiltration and tissue disorganization in the wound bed than gauze, but less than foam. Studies are now emerging showing that the amount and character of granulation tissue formed under foam and gauze differ.^34^^,^^35^ The present findings are in line with previous studies showing that the granulation tissue formed under foam is thick, while that under gauze is thinner but denser.^19^^,^^34^^,^^35^ The results of this study show that the use of pathogen-binding mesh leads to the formation of granulation tissue with properties between those of granulation tissue produced when using foam and gauze.

The reason for the morphological difference and leukocyte infiltration in the wound bed tissue underlying pathogen binding mesh, foam, and gauze cannot be deduced from this study. It may be that either the chemical or the geometrical properties of the wound filler plays a role. One possible mechanism could be that the wound filler causes a “foreign body reaction.” Leukocytes would then release cytokines to promote granulation tissue formation. The first step in the process of granulation tissue formation is disorganization of the tissue as the cells turn into fibroblasts.^44^ Disorganization was seen in our sliced sections of the wound bed, with disruption of the contacts between the cells and differences in cell sizes. Once fibroblasts are formed, they create granulation tissue. The fact that there is a difference in leukocyte infiltration and disorganization in the tissue between the 3 different wound fillers used for the study may relate to the difference in granulation tissue properties and wound healing in the clinical situation. It is well known that in wounds treated with foam the granulation tissue may be thick, hypertrophic, and fragile, while in wounds treated with gauze the granulation tissue is more dense and stable.

The wound filler used in NPWT is chosen to suit specific wounds.^36^ Thick granulation tissue is beneficial for fast wound healing, but may lead to problems such as fibrosis, scarring and, contractures as the wound heals.^35^ Foam is thus suitable for wounds that benefit from thick granulation tissue and where scarring does not pose a problem, for example, in sternotomy wounds,^12^ or fasciotomy wounds in upper or lower limb compartment syndrome where contraction is beneficial,^45^ and in acute wounds with large tissue loss providing a bridging therapy.^3^^,^^4^ Gauze has become especially popular among plastic surgeons for wound-bed preparation before grafting^46^ and is the filler of choice when the cosmetic result is more important than the speed of wound healing, or in cases where scar tissue may restrict movement, for example, over joints. Pathogen-binding mesh produces a granulation tissue with characteristics between those formed with foam and gauze, providing clinicians with another wound filler in their efforts to obtain optimal healing effects.

### Microdeformation

The properties of the wound-filler interface determine many of the effects of NPWT on the wound bed as the tissue surface is stimulated by the structure of the wound dressing. The interaction between the filler and the wound bed has been described in detail for foam and gauze.^19^^,^^47^ Histological examination of cross-sections of the wound bed in this study showed that the use of pathogen-binding mesh, foam, and gauze all resulted in an undulating wound bed surface, and that small tissue blebs or “mushrooms” were drawn into the pores of the foam dressing and between the threads of the gauze. This microdeformation is thought to result in shearing forces at the wound-dressing interface that affect the cytoskeleton and initiate a series of biological effects, including the stimulation of angiogenesis^48^^,^^49^ and the promotion of granulation tissue formation, leading to wound healing.^16^

### Ingrowth and the force needed to remove the wound filler

Little force was required to remove pathogen-binding mesh and gauze, while considerable force was needed to remove the foam. This is probably due to the ingrowth of tissue into the foam, but not into pathogen-binding mesh or gauze, as seen when examining biopsy sections from the wound bed in a light microscope. A number of complications are associated with tissue ingrowth into foam. First, the patient may experience pain during dressing changes as the ingrown tissue is torn away from the wound,^50^ requiring the administration of strong analgesics.^51^^,^^52^ Second, wound-bed disruption and mechanical tissue damage may arise as the foam is removed from the wound bed during dressing changes. Third, pieces of foam may become stuck in the wound bed and, if not removed, will act as foreign bodies that may impede wound healing. It is therefore common that a nonadherent wound contact layer is placed between the wound bed and the wound filler, when the clinician anticipates such complications.^53^^,^^54^ A low-adherence wound contact layer may also be placed over vulnerable structures such as blood vessels or nerves.^54^ The mechanism governing tissue ingrowth into foam is probably related to the interaction between tissue and dressing at a microscopic level.^17^ The differences in ingrowth observed in this study are probably due to differences in the physical properties of the dressings.

### Ease of application

Gauze is often used because of its mouldability and ease of application to large and irregular wounds. he use of gauze in NPWT has been described by Jeffery et al when treating wounds resulting from land mines and other explosive devices in military personnel.^55^ Pathogen-binding mesh is also a woven material, and the application technique is similar to that of gauze.

### Pathogen-binding mesh for managing wound infection during NPWT

Pathogen-binding mesh provides an interesting alternative wound filler because it is known to bind and inactivate bacteria and fungi.^37^ Furthermore, there is clear evidence that NPWT results in a shift in the bacterial community toward biofilm-producing organisms, for example, *S. aureus* and *S. epidermidis* during NPWT,^27^^,^^28^^,^^30^ which are the kind of bacteria that pathogen-binding mesh is known to counteract.^38^^,^^39^ The use of pathogen-binding mesh as a wound filler in NPWT may be especially beneficial when infection causes difficulty in wound healing.

## CONCLUSIONS

Pathogen-binding mesh as a wound filler in NPWT leads to the formation of a significant amount of granulation tissue in the wound bed, more than that for gauze, without the problems of ingrowth of the wound bed into the wound filler, as in the case with foam. Furthermore, pathogen-binding mesh has the advantage of being antimicrobial and easy to apply, like gauze, and thus constitutes an interesting alternative wound filler in NPWT.
